# Effects of Ovarian Hormones and Oral Contraceptive Pills on Cardiac Vagal Withdrawal at the Onset of Dynamic Exercise

**DOI:** 10.1371/journal.pone.0119626

**Published:** 2015-03-18

**Authors:** André L. Teixeira, Plinio S. Ramos, Lauro C. Vianna, Djalma R. Ricardo

**Affiliations:** 1 Maternity Hospital Therezinha de Jesus, Faculty of Medical and Health Sciences—SUPREMA, Juiz de Fora, MG, Brazil; 2 Postgraduate Program in Exercise and Sports Sciences, State University of Rio de Janeiro (UERJ), Rio de Janeiro, RJ, Brazil; 3 Faculty of Physical Education, University of Brasília, Brasília, DF, Brazil; Temple University, UNITED STATES

## Abstract

The purpose of this study was to investigate the effects of the ovarian hormones and the use of oral contraceptive pills (OCP) on cardiac vagal withdrawal at the onset of dynamic exercise. Thirty physically active women aged 19–32 years were divided into two groups: OCP users (n = 17) and non-OCP users (n = 13). Participants were studied randomly at three different phases of the menstrual cycle: early follicular (day 3.6 ± 1.2; range 1–5), ovulatory (day 14.3 ± 0.8; range 13–16) and midluteal (day 21.3 ± 0.8; range 20–24), according to endogenous (in non-OCP users) or exogenous (in OCP users) estradiol and progesterone variations. The cardiac vagal withdrawal was represented by the cardiac vagal index (CVI), which was obtained by the 4-s exercise test. Additionally, resting heart rate, systolic (SBP) and diastolic blood pressure (DBP) were obtained. The CVI was not significantly different between the three phases of the menstrual cycle in either the non-OCP users (early follicular: 1.58 ± 0.1; ovulatory: 1.56 ± 0.1; midluteal: 1.58 ± 0.1, *P* > 0.05) or OCP users (early follicular: 1.47 ± 0.1; ovulatory: 1.49 ± 0.1; midluteal: 1.47 ± 0.1, *P* > 0.05) (mean ± SEM). Resting cardiovascular responses were not affected by hormonal phase or OCP use, except that the SBP was higher in the OCP users than non-OCP users in all phases of the cycle (*P* < 0.05). In summary, our results demonstrate that cardiac vagal withdrawal at the onset of dynamic exercise was not impacted by the menstrual cycle or OCP use in physically active women.

## Introduction

Women who experience early menopause are at increased risk of cardiovascular disease, including coronary artery disease and hypertension, compared with age-matched premenopausal women [1,2]. This low incidence of cardiovascular disease in premenopausal women has been attributed to the protective effects of endogenous female hormones, specifically estrogen [3,4]. In premenopausal women, the ovarian hormones fluctuate monthly with the menstrual cycle (MC); specifically, the early follicular phase is characterized by low levels of estradiol and progesterone, ovulatory phase occurs at the peak of estrogen, and the midluteal phase is characterized by high concentration of progesterone and, to a lesser extent, estrogen [5]. In addition, the use of oral contraceptive pills (OCP) is a strategy often used by women to prevent pregnancy, in which a constant dosage of female hormones attenuates these cyclical variations [6]. Importantly, the impact of ovarian hormones fluctuation as well as the OCP on the neural control of the heart remains to be further explored.

Several studies have examined the impact of fluctuations in female sex hormones across the MC on the autonomic control of the heart at rest. However, it remains unclear as to how the autonomic nervous system is impacted by the ovarian hormones fluctuations with equivocal results being reported. For example, Tanaka et al. [7] showed that the baroreflex-mediated control of heart rate (HR) is altered during different phases of the MC. Bai et al. [8] showed, through nonlinear properties of HR variability, that vagal activity predominates in the early follicular phase and that sympathetic activity is higher in the midluteal phase of the cycle. However, Leicht et al. [9] reported that the normal cyclic variations in hormone levels during the MC were not associated with changes in cardiac autonomic control, as assessed by HR variability. In addition, Teixeira et al. [5] found that different phases of the MC did not modify the resting HR in healthy women independent of the use of OCP. The reason for these contradictory findings is not clear but additional work is needed to further our understanding on the impact of ovarian hormones fluctuation on autonomic control of the heart, specifically under conditions where the heart is challenged, such as dynamic exercise.

During the transition from rest-to-exercise, the rapid HR increase is predominantly mediated by vagal withdrawal, and afterward, with an increase in exercise intensity, the HR accelerates via an increase in sympathetic activity [10,11]. Matsuo et al. [12] demonstrated that HR responses at the onset of voluntary exercise and passive movement were not altered by the MC in seven healthy women, as measured by ΔHR during the 20-s of exercise. Although this suggests lack of MC effect on the neural control of HR, a potential caveat is that this study has used only the follicular and luteal phases of the MC and ignored the ovulatory phase where there are higher levels of estradiol and low levels of progesterone. Furthermore, these authors did not aim to isolate the vagally-mediated HR response, since HR is also modulated by sympathetic activity in exercise of this duration [13,14]. To assess the relative contribution of vagal activity during the rest-to-exercise transition, Araújo et al. [15] proposed a 4-s exercise test (4sET) protocol, in which predominantly vagal activity at rest is suddenly withdrawn by fast, unloaded cycling exercise. The cardiac vagal withdrawal was represented by the cardiac vagal index (CVI) measured through the ratio between the longest and shortest RR interval during the onset of exercise. Although the 4sET is pharmacologically validated [16] and has high intra and interday reliability [17], the influence of the MC and OCP in this context needs to be further studied.

Given these considerations, we hypothesized that the cardiac vagal withdrawal would vary with the endogenous female hormones throughout the MC and that these cyclical changes in autonomic control would be blunted by the administration of OCP. Therefore, the purpose of this study was to assess the effects of endogenous variations in ovarian hormones in non-OCP users, and the administration of OCP across the MC in OCP users on cardiac vagal withdrawal at the onset of dynamic exercise in healthy young women.

## Materials and Methods

### Ethics statement

All study procedures were approved by the local institutional research committee (protocol number 276.778) in accordance with the Declaration of Helsinki of the World Medical Association Ethics Code. All subjects participated in the present study voluntarily, receiving no financial incentive. Participants were informed that they could withdraw at any time. Each subject read and signed a specific informed consent form.

All data are only available upon request to the senior author (legal guardian of the data). This option follows the local laws and ethical regulations that apply to confidentiality of clinical/medical data.

### Subjects

A total of 30 healthy women aged 19–32 years (mean ± SEM: 24.7 ± 0.65 years) were enrolled in this study. Participants were divided into two groups: non-OCP users (n = 13) and OCP users (n = 17). A detailed medical history was obtained from all participants, and they underwent a standard physiological examination. They were confirmed to have no heart disease. As inclusion criteria, subjects had to be eumenorrheic, asymptomatic, nonsmokers, normotensive, nondiabetic, nonobese (body mass index—BMI—falling in the normal weight category of 18.5–24.9 kg/m^2^), physically active (habitual physical activity for at least 6 consecutive months with a minimum frequency of 3 days per week in ≥30-min sessions), and non-users of any medications that could interfere with the experimental procedures. Participants also reported no intention to become pregnant within the next two months.

## Measurements

### Menstrual phases

Initially, each participant recorded the timing of their MC in a MC history questionnaire covering the previous two months. Participants were evaluated during three different phases of the MC: early follicular (day 3.6 ± 1.2; range 1–5), ovulatory (day 14.3 ± 0.8; range 13–16) and midluteal (day 21.3 ± 0.8; range 20–24). The timing of the menstrual phases was confirmed by analysis of the blood levels of estradiol and progesterone. For this assessment, a 5-ml blood sample was collected from the antecubital vein and centrifuged at 5000 rpm for 5 min at 4°C, and serum ovarian hormones were measured by an electrochemiluminescence technique (Architect Plus, I 2000, Abbot Diagnostics, USA). Women entered the study at different phases of the MC. 12 women (6 non-OCP users) initiated the study procedures in the early follicular phase; 10 women (4 non-OCP users) in the ovulatory phase; and 8 women (3 non-OCP users) in the midluteal phase of the MC.

### Cardiac vagal withdrawal

For assessment of cardiac vagal withdrawal, we used the 4sET. Originally proposed by Araújo et al. [15], the 4sET is pharmacologically validated [16] and has high within-day and between-day reliability [17] for the isolated assessment of the integrity of cardiac vagal activity through the analysis of HR at the onset of dynamic exercise. This test is based on the concept that the latency of the sympathetic branch is longer than parasympathetic branch; therefore, at the onset of dynamic exercise, there is an almost instantaneous vagal withdrawal via a neurogenic reflex that is followed by sympathetic stimulation [18]. Previous studies have described the 4sET in detail [18–27]. Briefly, the 4sET consists of unloaded pedaling as fast as possible on a cycle ergometer (Imbramed, CG—04, Brazil) from the fourth to the eighth seconds of a 12-s maximal-inspiratory apnea. The subject remains seated on the cycle ergometer, and after the HR stabilizes, the verbal commands of four evaluators guide the participant’s actions to be sequentially performed at 4-s intervals, as follows: 1) a fast maximal inspiration, primarily through the mouth, 2) pedaling as fast as possible, 3) sudden cessation of pedaling, and 4) expiration. A continuous recording of a single electrocardiogram (ECG) lead, usually CC_5_ or CM_5_, was obtained continuously during the test. All cardiovascular variables were sampled at 1,000 Hz and stored for offline analysis (PowerLab 4/25T and Lab Chart Pro 7 software; ADInstruments, Australia).

**Fig 1:**
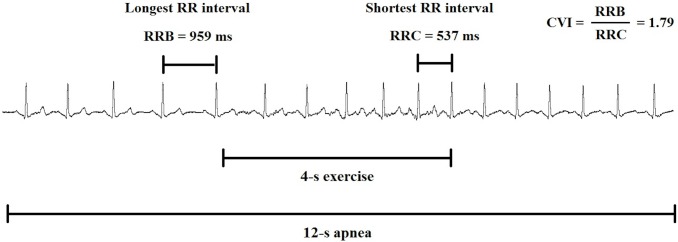
A typical ECG tracing during the 4-s exercise test in one subject of the sample. CVI: cardiac vagal index.

To quantify CVI, the duration of two RR intervals was measured: the longest RR interval (*i*.*e*., either the interval obtained immediately before the onset of exercise or the first interval after the onset of exercise; RRB) and the shortest RR interval during the 4sET (generally, the last RR interval; RRC). Two maneuvers were typically carried out, with a dimensionless index (the greatest ratio between the aforementioned intervals obtained during both 4sET maneuvers) being chosen to represent the CVI. Fig. 1 presents the calculation of CVI in a representative ECG tracing during the 4sET in one volunteer of the current study.

## Study Protocol

Subjects were evaluated during three different phases of the MC. The subjects were asked to refrain from consuming caffeine/alcohol and from engaging in physical exercise for 6 and 24 h, respectively, prior to the tests. To avoid potential diurnal variations, subjects were always tested at the same time of day and in the same quiet, temperature-controlled room (24°C).

Initially, blood samples were obtained for hormonal analysis and confirmation of menstrual phases. The participants’ body weight was assessed using a digital weighing scale (Welmy, Brazil). Height was determined using a stadiometer with millimeter precision (Sanny, Brazil). Then, BMI was calculated as weight in kilograms divided by the square of height in meters (kg/m^2^). Resting HR was obtained by a one-lead (CC_5_ or CM_5_) ECG using a PowerLab system (PowerLab 4/25T and Lab Chart Pro 7 software; ADInstruments, Australia). Systolic (SBP) and diastolic blood pressure (DBP) were obtained by the auscultatory method (BD, Brazil). Participants were asked to be in the supine position in a relaxed state for 20 min. Measurements were determined during the final 10-min period. An initial 10-min period was utilized to ensure the attainment and stabilization of the HR. After resting measurements, subjects were submitted to the 4sET to assess CVI.

### Statistical analysis

The Shapiro-Wilk normality test and Levene’s test of homogeneity of variance were used to analyze the normality of the distribution of data. All variables presented a normal distribution and equal variance. Significant differences between groups in descriptive data were assessed by the independent sample *t* test. A 2 x 3 (groups x menstrual phases) repeated-measures ANOVA was used to compare the hormone levels, cardiovascular responses and CVI between groups throughout the MC. We then tested the sphericity of the data and used Bonferroni’s post hoc test to detect differences when necessary. Effect size (ES) for each physiological variable was calculated using the partial Eta squared. Threshold values for ES were 0.02 (small), 0.13 (moderate), 0.26 (large) [28,29]. We used either Prism (version 5.01; GraphPad, USA) or SPSS (version 19; SPSS, USA) to perform all calculations and generate figures. The significance level adopted was *P* < 0.05.

## Results

The descriptive data of the subjects are presented in Table 1. No significant differences were observed between the groups in any descriptive measurement (*P* > 0.05).

**Table 1:** Descriptive data of the subjects.

	No OCP (n = 13)	OCP (n = 17)	*P*
**Age (y)**	25.2 ± 1.0	24.4 ± 0.8	0.55
**Weight (kg)**	57.5 ± 1.8	59.1 ± 1.3	0.46
**Height (m)**	1.62 ± 0.02	1.64 ± 0.01	0.26
**BMI (kg/m^2^)**	22.0 ± 0.5	22.0 ± 0.3	0.99
**Menarche (y)**	12.5 ± 0.3	12.5 ± 0.2	0.85

Values presents in mean ± SEM. No OCP: non-OCP users. OCP: OCP users. BMI: body mass index. *P*: level of significance (p < 0.05).

**Fig 2:**
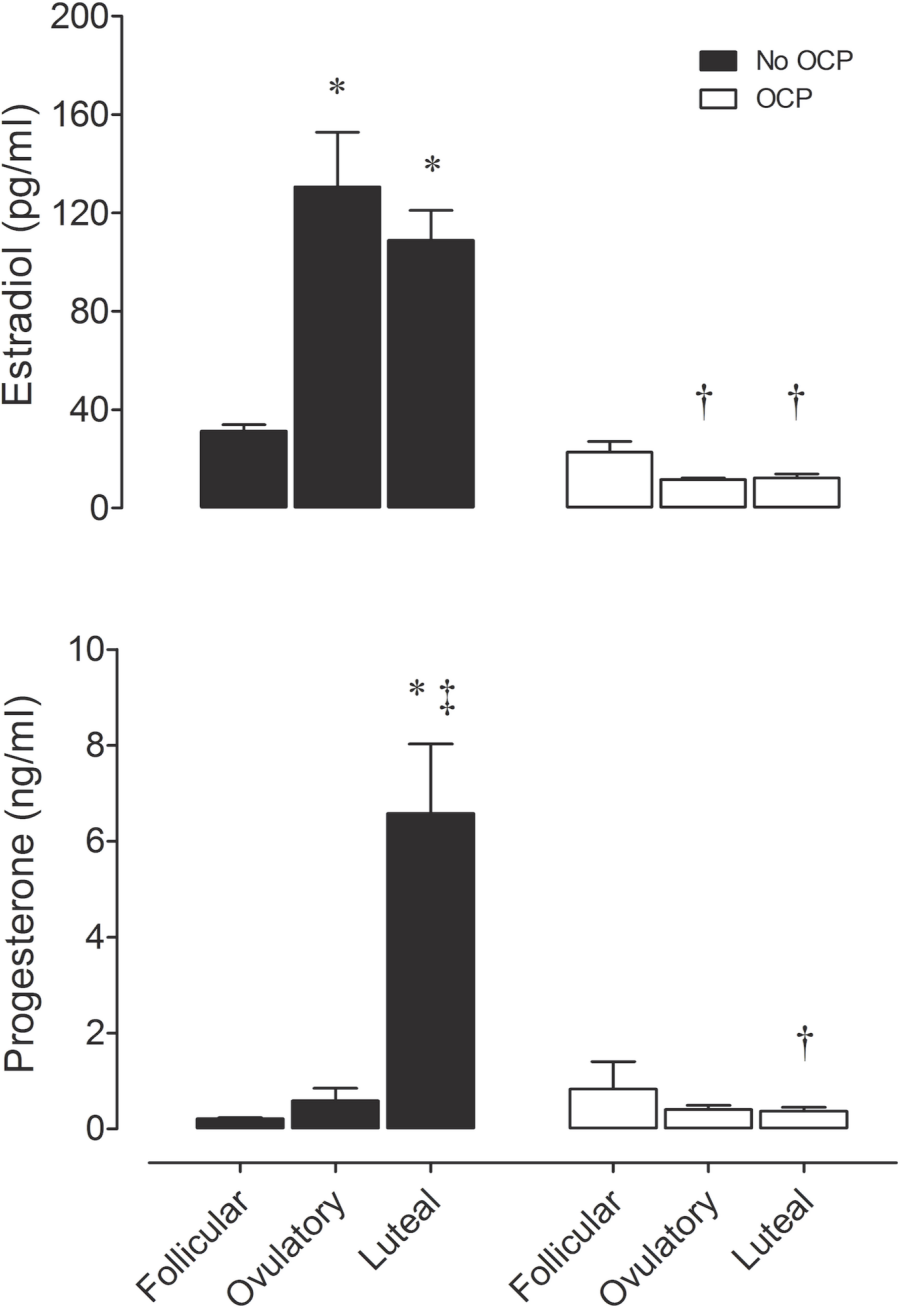
Levels of estradiol (above) and progesterone (below) between non-OCP users (No OCP) and OCP users (OCP) during three different phases of the cycle. * Significant difference (p < 0.001) to early follicular phase. † Significant difference (p < 0.001) between groups in same phase of the MC. ‡ Significant difference (p < 0.001) to ovulatory phase.

Fig. 2 presents resting ovarian hormone concentrations across the MC. In non-OCP users, plasma estradiol concentrations were significantly higher during ovulatory (mean ± SEM: 130.6 ± 22.2 pg/ml) and midluteal (mean ± SEM: 108.8 ± 12.3 pg/ml) phases compared with early follicular phase (mean ± SEM: 31.3 ± 2.6 pg/ml) (*P* < 0.05), whereas plasma progesterone level during midluteal phase (mean ± SEM: 6.577 ± 1.45 ng/ml) was significantly higher than early follicular (mean ± SEM: 0.215 ± 0.02 ng/ml) and ovulatory (mean ± SEM: 0.585 ± 0.26 ng/ml) phases of the MC. In OCP users, both plasma estradiol (early follicular: 22.8 ± 4.2; ovulatory: 11.6 ± 0.7; midluteal: 12.3 ± 1.5 pg/ml; *P* > 0.05) (mean ± SEM), and progesterone (early follicular: 0.829 ± 0.58; ovulatory: 0.406 ± 0.09; midluteal: 0.371 ± 0.08 ng/ml; *P* > 0.05) (mean ± SEM) concentrations were similar in all phases of MC. In addition, estradiol levels in non-OCP users was significantly higher in both ovulatory and midluteal phases, and progesterone levels was higher in midluteal phase of the cycle compared with OCP users (*P* < 0.05).

**Fig 3:**
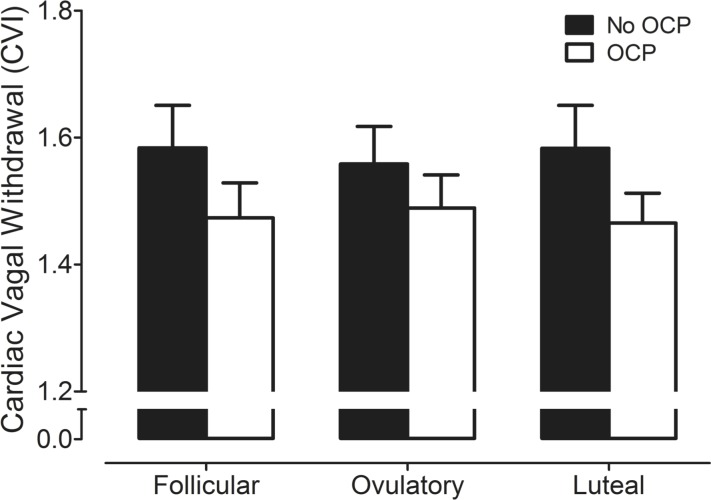
Cardiac vagal withdrawal represented by cardiac vagal index (CVI) in non-OCP users (No OCP) and OCP users (OCP) across the menstrual cycle.

Fig. 3 shows that the CVI was not significantly different across the MC in either non-OCP users (early follicular: 1.58 ± 0.1; ovulatory: 1.56 ± 0.1; midluteal: 1.58 ± 0.1, *P* > 0.05) or OCP users (early follicular: 1.47 ± 0.1; ovulatory: 1.49 ± 0.1; midluteal: 1.47 ± 0.1, *P* > 0.05). In addition, there were no significant differences in CVI between groups during the three different phases of the MC (*P* > 0.05; partial Eta squared: menstrual cycle = 0.002; group = 0.054; interaction = 0.028).

The resting cardiovascular responses were presents in Table 2. HR and DBP did not vary according to MC phase or OCP use (*P* > 0.05). SBP was not impacted by different phases of the MC in both non-OCP users and OCP users (*P* > 0.05). However, SBP values were significantly higher in OCP users than non-OCP users in all phases of the MC (*P* = 0.004).

**Table 2:** Resting cardiovascular values across the menstrual cycle in non-OCP users (No OCP) and OCP users (OCP).

		No OCP	OCP	Partial Eta squared		
				MC	Group	Interaction
**HR rest (bpm)**	**Follicular**	66.2 ± 2.1	70.7 ± 2.8	0.087	0.008	0.085
	**Ovulatory**	71.8 ± 2.4	70.3 ± 2.0			
	**Luteal**	70.4 ± 2.8	71.8 ± 2.4			
**SBP (mm/Hg)**	**Follicular**	102.3 ± 2.0	111.1 ± 2.4*	0.014	0.182	0.022
	**Ovulatory**	103.5 ± 2.2	108.5 ± 2.0*			
	**Luteal**	102.2 ± 1.5	108.0 ± 3.0*			
**DBP (mm/Hg)**	**Follicular**	68.0 ± 2.0	71.3 ± 1.6	0.063	0.067	0.011
	**Ovulatory**	67.1 ± 2.0	68.4 ± 1.5			
	**Luteal**	65.4 ± 1.7	68.6 ± 1.6			

Values presents in mean ± SEM. HR: heart rate, SBP: systolic blood pressure, DBP: diastolic blood pressure. MC: menstrual cycle.

* Significant difference (p < 0.05) between groups.

## Discussion

The MC is characterized by rhythmic variation in the secretion of female hormones and corresponds to changes in the sexual organs and other physiological responses. The purpose of this study was to assess the effects of the ovarian hormones and OCP on cardiac vagal withdrawal at the onset of dynamic exercise. The major findings are the following: 1) CVI was not affected by endogenous ovarian hormones during the normal MC in non-OCP users, and the administration of OCP with a constant exogenous dosage of sex hormones did not change the cardiac vagal withdrawal; and 2) resting cardiovascular responses, including HR and blood pressure were not affected by the MC or OCP, with the exception of SBP which was significantly higher in OCP users compared with non-OCP users in all phases of the MC.

Although several methods are available to assess cardiac parasympathetic activity, in the present study we used the 4sET. This test is based on the concept that the latency of the sympathetic branch is longer than that of the parasympathetic branch; therefore, at the onset of dynamic exercise, an almost instantaneous vagal withdrawal occurs via a neurogenic reflex that is followed by sympathetic stimulation [18]. The 4sET has been pharmacologically validated in 15 subjects who had a complete abolition of 4-s heart rate rest-exercise transients under atropine, while large amounts of intravenous propanolol did not influence this response (the ratio between cardiac cycle interval durations measured at pre-exercise and post-fast unloaded cycling) [16]. Furthermore, Araújo et al. [17] tested the reliability of the 4sET. The interday reliability was assessed prospectively from 15 asymptomatic subjects submitted to 4sET for five consecutive days. To determine CVI intraday reliability, in one of the five days, randomly selected, nine 4sET consecutive trials were made. CVI presented high interday and intraday reliability (r_i_ = 0.77; 95%CI = 0.49 to 0.92 and r_i_ = 0.92; 95%CI = 0.84 to 0.97, respectively) [17]. Previous studies have shown that the CVI magnitude is not dependent on active or passive execution [24], on whether the exercise is undertaken with the lower or upper limbs [25], or even without an ergometer usage, in the orthostatic position [26]. This simple and highly reliable protocol has been used in various physiological and clinical studies [15–27].

The results of the present study are similar to those of Matsuo et al. [12], who verified the influence of the MC on the initial HR transient during the onset of exercise. They evaluated seven non-OCP users through voluntary exercise and passive movement of knee extension for 20 s. To quantify cardiac autonomic control, they used ΔHR, calculated as the change in the absolute value of HR. In agreement with our results, they reported that HR increased similarly during exercise in both the follicular and luteal phases of the MC in non-OCP users. However, authors not used the ovulatory phase of the cycle in which estrogen concentrations were higher and progesterone levels were low. These results suggest that during the initial transient phase of exercise, no differences exist in the autonomic nervous system during the different phases of the MC. Nevertheless, other authors, who used different methodological designs, reported that the MC may change cardiac autonomic control, as discussed below.

Resting HR and blood pressure results, were not affected by the phase of the cycle in either non-OCP or OCP users. However, SBP was higher in OCP users than non-OCP users in both menstrual phases. OCP users have higher cardiovascular disease rates than non-OCP users [6], and our results corroborate this finding, as our healthy young OCP users already had higher SBP values than the non-OCP users. This fact may be due to the protective effects of female sex hormones on the cardiovascular system [3,4] because in non-OCP users, estrogen and progesterone levels fluctuated throughout the MC, and the use of OCPs attenuates these cyclical variations (see Fig. 2).

Previous studies with different methodological designs have assessed the effects of the MC on cardiac autonomic control. Some, but not all, studies have found that endogenous estradiol and progesterone fluctuations during the MC are associated with changes in sympathetic and parasympathetic activities. Sato et al. [30] observed, through a power spectral analysis of HR variability, that sympathetic nervous system activities were predominant in the luteal phase compared with the follicular phase of the MC. Tanaka et al. [7] demonstrated that baroreflex control of HR was altered during the regular MC, and that estradiol was associated with cardiovagal modulation in 15 healthy women. McKinley et al. [31] found that resting HR was lower and HR variability was higher during the follicular phase than the luteal phase of the MC. In addition, Bai et al. [8] demonstrated through a HR variability assessment that the high-frequency components decreased from the follicular phase to the luteal phase, while the low-frequency components and the low and high-frequency ratio increased. According to these authors, the follicular phase is characterized by enhanced vagal activity and the luteal phase is characterized by enhanced sympathetic activity.

In contrast to the aforementioned results, other authors have found no significant differences in the autonomic control of HR throughout the MC. For example, Leicht et al. [9] showed that the normal cyclic variations in endogenous female hormone levels that occurred during the MC in non-OCP users were not significantly associated with changes in cardiac autonomic control as measured by HR variability. Teixeira et al. [5] compared non-OCP users and OCP users throughout the MC and found no significant differences between groups or within groups in resting HR during three different phases of the MC (follicular, ovulatory and luteal). Additionally, Middlekauf et al. [32] compared baroreceptor and non-baroreceptor control during the follicular and luteal phases of the MC in non-OCP and OCP users. The authors used various protocols to analyze baroreceptor and non-baroreceptor control, including microneurography, baroreflex activation and deactivation, and the cold pressor test, and the results showed a significant difference only in sympathetic nerve activity, which was lower during the follicular (low ovarian hormones) compared with the luteal (high ovarian hormones) phase in non-OCP users but was not different between phases in OCP users. No significant differences were obtained in all others variables. These studies are in agreement with our observation that no differences were obtained between groups or within groups in CVI and resting cardiovascular responses.

The reason for the discrepancies between various studies is not clear, but they may be attributed to the different methods used to assess cardiac autonomic control. Potentially, a subtle and currently unrecognized difference in patient populations may underlie these discrepant findings [32]. Another factor that can explain the discrepancies between previous studies and the present study is the inclusion of only physically active women in the present study, as exercise can modify sympathetic and parasympathetic cardiac autonomic control [33]. One factor that should be considered during the analysis of the studies is to estimate the magnitude of the differences. Various methods have been described to estimate the magnitude of an ES and partial Eta squared has been shown to be the most common and appropriate method. Based on the Bayesian analysis, we must consider our results physiologically meaningful.

It is important to note the limitations of this study, such as the size of the study sample and the potential for type II error. From a more conservative standpoint the study was potentially underpowered to detect an interaction between groups and phases of the MC. In OCP users, our volunteers used several different types of OCP. Although the dosages may have been different, all OCP were combination pills, containing both synthetic estrogen and progesterone, and previous studies have used the same methodological design [5,32]. In addition, although we used only physically active women, the level of physical fitness of each subject was not controlled. Future research should be conducted with more definitive determination of the OCP, and the inclusion of other factors such as physical fitness level and clinical status for future comparison of results and better clarification on this issue.

Previous studies have shown the protective effects of estrogen on the cardiovascular system [3,4]. Estrogen has both rapid vasodilatory effects, including enhanced nitric oxide systhesis, and longer-term actions that inhibit the response to vascular injury and prevent atherosclerosis [3]. In this context, Weissman et al. [34] demonstrated that the acute increase in estrogen level during the ovulation induction protocol is associated with vagal activation in young women. The findings in the present study do not confirm a role for endogenous or exogenous female sex hormone fluctuations in CVI at onset of exercise or in cardiovascular responses during rest in non-OCP or OCP users.

## Conclusions

In summary, the results of this study provide preliminary evidence that the cardiac vagal withdrawal at the onset of dynamic exercise and the resting cardiovascular variables, including HR and blood pressure are not affected by the MC and that these findings are similar between non-OCP and OCP users.
